# What influences the public’s willingness to report health insurance fraud in familiar or unfamiliar healthcare settings? a cross-sectional study of the young and middle-aged people in China

**DOI:** 10.1186/s12889-023-17581-9

**Published:** 2024-01-02

**Authors:** Jinpeng Xu, Ting Zhang, Hongyu Zhang, Fangmin Deng, Qi Shi, Jian Liu, Fangting Chen, Jingran He, Qunhong Wu, Zheng Kang, Guomei Tian

**Affiliations:** 1 School of Health Management, Harbin Medical University, Harbin, Heilongjiang China; 2 Department of Nuclear Medicine, The Fourth Hospital of Harbin Medical University, Harbin, Heilongjiang China

**Keywords:** Health Insurance Fraud, Willingness to Report, Healthcare setting, Familiar or not

## Abstract

**Introduction:**

Young and middle-aged people are important participants in the fight against health insurance fraud. The study aims to investigate the differences in their willingness to report health insurance fraud and the factors influencing it when it occurs in familiar or unfamiliar healthcare settings.

**Methods:**

Data were obtained from a validated questionnaire from 828 young and middle-aged people. McNemar’s test was used to compare the public’s willingness to report under the two scenarios. Chi-square tests and multiple logistic regression analysis were used to analyze the determinants of individuals’ willingness to report health insurance fraud in different scenarios.

**Results:**

Young and middle-aged people were more likely to report health insurance fraud in a familiar healthcare setting than in an unfamiliar one (McNemar’s χ²=26.51, *P* < 0.05). Their sense of responsibility for maintaining the security of the health insurance fund, the government’s openness about fraud cases, and the perception of their ability to report had significant positive effects on the public’s willingness to report in both settings (*P* < 0.05). In a familiar healthcare setting, the more satisfied the public is with government measures to protect whistleblowers, the more likely they are to report (*OR* = 1.44, *P* = 0.025). Those who perceive the consequences of health insurance fraud to be serious are more likely to report than those who perceive the consequences to be less serious (*OR* = 1.61, *P* = 0.042).

**Conclusion:**

Individuals are more likely to report health insurance fraud in familiar healthcare settings than in unfamiliar ones, in which their awareness of the severity of the consequences of health insurance fraud and their perceived risk after reporting it play an important role. The government’s publicizing of fraud cases and enhancing the public’s sense of responsibility and ability to maintain the safety of the health insurance fund may be a way to increase their willingness to report, regardless of whether they are familiar with the healthcare setting or not.

## Introduction

Health insurance fraud, which is the intentional deception or misrepresentation by a person or entity to obtain unauthorized benefits [1], is a significant part of the crisis in healthcare systems around the world and has been identified as a global challenge and illegal activity [2, 3]. Several studies have shown that health insurance fraud leads to a significant increase in the cost of national health insurance programs and is one of the main causes of inefficiencies in the operation of health insurance funds [4, 5]. In particular, in some high-income countries, 3–10% of annual healthcare expenditures are lost as a result of health insurance fraud, amounting to billions of dollars [6–9]. However, due to the unique characteristics of the health insurance industry and the complex and insidious of health insurance fraud, the supervision of health insurance funds is very difficult [10, 11].

The primary method of detecting health insurance fraud at present is manual detection by government-organized experts, supplemented by some machine-learning tools [12]. However, as a quasi-public good, health insurance funds deserve to be supervised by the public. The public has the natural advantages of wide distribution, strength, and quick detection, making public reporting one of the most important ways to combat health insurance fraud [13]. A study showed that 90% of health insurance fraud lawsuits in the United States between 1996 and 2005 came from whistleblowers [14]. The willingness of the public to report health insurance fraud is a prerequisite for the occurrence of reporting behavior [15, 16], and it is meaningful to study the willingness of the public to report health insurance fraud. In addition, due to a lack of understanding of relevant concepts, difficulty in changing mentalities, being satisfied with their retirement, and limited time and energy, the motivation and feasibility of older people’s participation in the governance of public affairs are not high [17, 18]. Therefore, despite the higher demand and utilization of healthcare services by older adults [19], increasing the willingness of young or middle-aged adults to report health insurance fraud may be a more effective way to conduct health insurance anti-fraud at present.

Nowadays, China’s basic health insurance coverage has stabilized at over 95%. With the increasing scale of health insurance financing ($423 billion in 2022), the importance of combating fraudulent insurance practices has become more prominent. Since health insurance fraud usually accompanies healthcare activities and occurs primarily at the point of obtaining healthcare services, regulating the behavior of healthcare organizations has become an important part of the supervision of the health insurance fund. A criminological study showed that when the public regularly travels to a particular location, it causes a change in the public’s familiarity with the particular environment, which in turn affects the public’s behavioral beliefs in that environment [20]. Influenced by the environment and interactions between individuals, the public is prone to detect health insurance fraud in the healthcare setting and they will identify themselves more strongly as potential whistleblowers [21], and their willingness to report will also be affected.

“Healthcare settings” represent a wide range of healthcare services and places where healthcare occurs, including but not limited to acute care hospitals, urgent care centers, rehabilitation centers, nursing homes, and other long-term care facilities, specialty outpatient services, and outpatient surgery centers [22]. Based on this, our study defines the healthcare providers frequently visited by the public as familiar healthcare settings and hypothesizes that familiar or unfamiliar healthcare settings may have an impact on the public’s willingness to report health insurance fraud. To the best of our knowledge, few scholars have conducted relevant studies in the field of health insurance anti-fraud. Therefore, this paper will focus on analyzing the differences in the public’s willingness to report health insurance fraud when it occurs in familiar or unfamiliar healthcare settings and compare the factors that influence their willingness to report in these two settings.

Numerous empirical studies have shown that the Theory of Planned Behavior (TPB) has good explanatory and predictive power for individual behavioral intentions [23–25]. Nonetheless, the theory’s assumptions may still be incomplete, leading to a need for improvement in its explanation of behavioral intentions and actual behavior [26]. Many scholars have also identified this shortcoming and introduced perceived consequences as a predictor variable to improve the predictive power of TPB for individual behavioral intentions [27]. The study of Triandis strongly confirmed the relationship between consequence perception and individual behavioral intention [28]. The study also found that people’s perceptions of the event itself were closely related to their behavioral intentions [29, 30].

In summary, this study will explore the introduction of two predictor variables based on the TPB, namely, an individual’s perception of the consequences of health insurance fraud (PCONSE) and an individual’s perception of risk after reporting health insurance fraud (PRISK), to construct an extended theoretical model of planned behavior that examines the factors that influence public’s willingness to report health insurance fraud in both familiar and unfamiliar healthcare settings. We aim to provide an in-depth understanding of the public’s behavioral intentions in the face of unreasonable behavior and to provide a basis to manage unreasonable behavior in various fields such as health insurance, food safety, and environmental protection.

## Methods

### Study design and participants

#### Sampling technique

A cross-sectional quantitative survey was conducted in China between February 19, 2022, and September 13, 2022. Influenced by COVID-19, large-scale face-to-face surveys are difficult to realize. Therefore, the study mainly conducted an online survey via the “Questionnaire Star platform” (a widely accepted online questionnaire survey platform in China). Before the formal survey, we conducted a pre-survey in which 60 questionnaires were collected using a convenience sampling method to improve our design and questionnaire quality.

According to the National Bureau of Statistics of China, the economic region was classified into four regions: Northeast, East, Central, and West [31]. Since the members of this study came from different provinces in China, to obtain a more credible sample and considering the accessibility of the participants, this study mainly collected data through the research members distributing the questionnaire links within their provinces and then collecting the data in a snowballing manner. Finally, Heilongjiang and Liaoning provinces were mainly selected as representatives in northeast China, Jiangsu and Shandong provinces in eastern China, Anhui and Shanxi provinces in central China, and Shaanxi and Guizhou provinces in western China. Respondents in this study were selected from residents of mainland China who were aged 18–60 years old and were eligible to participate anonymously. The introductory section of the questionnaire provided written informed consent before the response.

#### Quality control

Before the survey began, we explained to respondents in detail what health insurance fraud is, in particular, and made sure they knew what health insurance fraud is for healthcare providers. Since this study examines the public’s willingness to report on healthcare providers, healthcare insurance fraud referred to in this study mainly means the fraudulent behaviors adopted by healthcare providers to defraud the basic healthcare insurance fund, which mainly includes unreasonable charging behaviors, irregular diagnostic and therapeutic behaviors as well as fictitious healthcare service behaviors, and so on. Based on China’s policy regulations, healthcare providers can be considered to be committing health insurance fraud if they exhibit any or some of the behaviors in Table 1.

**Table 1:** Potential health insurance fraud by healthcare providers

Behavior type	Specific behaviors
Unreasonable charging behaviors	(1) Breaking down diagnostic and treatment items into multiple items for charging; (2) Duplicating charges for diagnostic and treatment items; (3) Charging over the stipulated charges; and (4) Crediting medical expenses that should be borne by individuals to the health insurance fund for payment.
Irregular diagnostic and therapeutic behaviors	(1) Breaking down a patient’s inpatient treatment process into two or more hospitalizations; (2) Admitting patients who do not meet the indications for hospitalization; (3) Excessive testing; and (4) Overprescribing and repetitive prescribing of medications
Fictitious healthcare services behaviors	(1) Forging or fictitious medical services; (2) Falsifying patient information; (3) Hospitalization under a registered name; (4) Falsifying medical documents and bills; (5) Inducing or assisting participants to seek medical treatment or purchase medicines under an impostor’s name or pretenses; (6) Providing false invoices for insured persons; and (7) Obtaining cash or purchasing non-medical items, such as cosmetics and daily necessities, for insured persons.

Meanwhile, according to the IP address recorded in the questionnaire, each participant could only answer once. If a questionnaire was completed in more than 10 min, which was the shortest time our team tested to complete the questionnaire, and logically answered two logical questions, it was judged valid and included in the analysis, otherwise, it was removed. In general, 900 people completed the questionnaire, screening 837 valid data, with a valid return rate of 93%. After excluding samples under 18 years of age and 60 years of age and above, a total of 828 study participants were included in the study.

### Theoretical framework

**Fig. 1:**
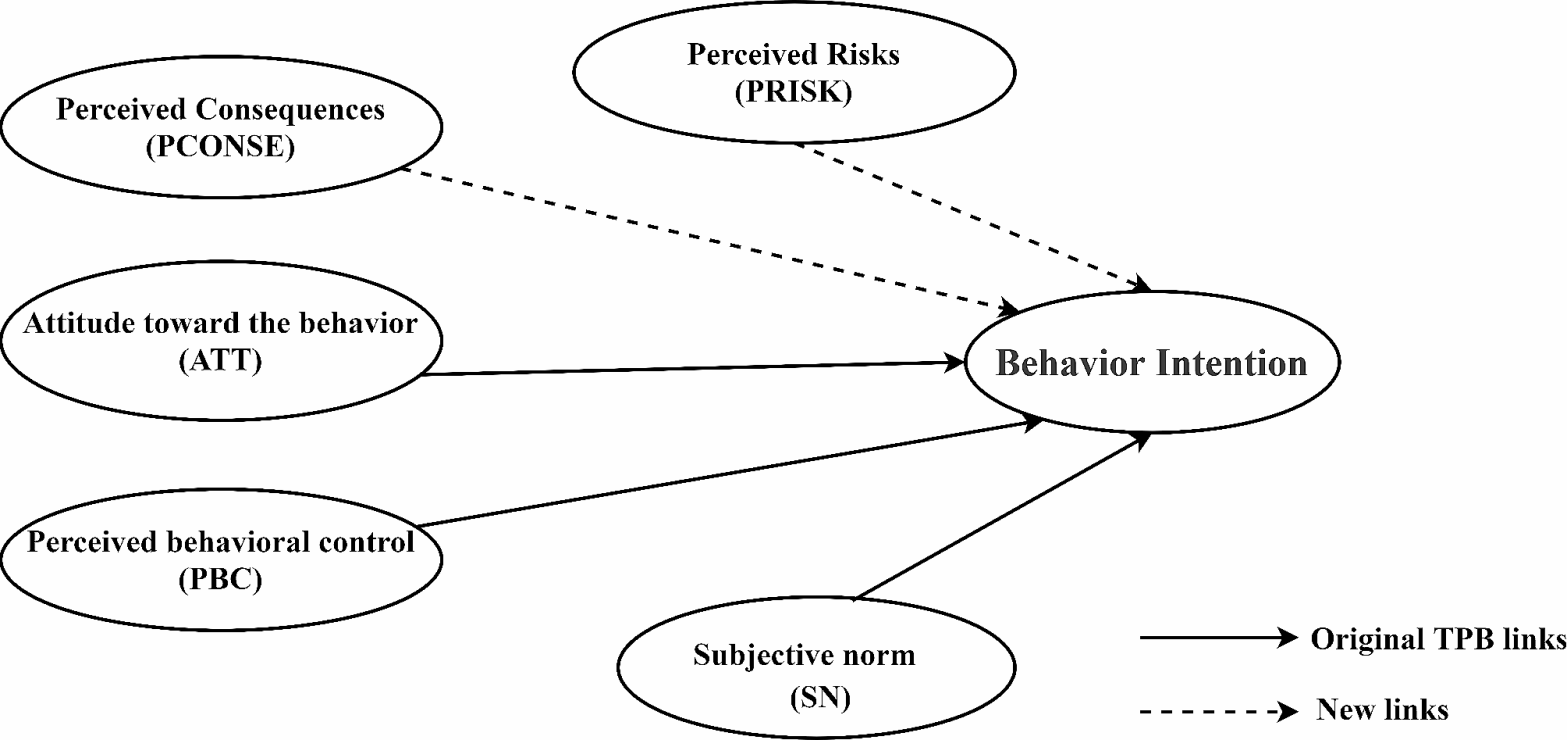
Extending the Theory of Planned Behavior

This study examines what factors influence the public’s willingness to report health insurance fraud based on the framework of an extended Theory of Planned Behavior model. TPB proposes that individual behavioral intentions are controlled by a combination of three factors: Attitudes toward the Behavior (ATT), Subjective Norms (SN), and Perceived Behavioral Control (PBC) [32, 33]. In our study, ATT refers to respondents’ positive or negative attitudes toward public participation in reporting health insurance fraud. SN refers to the social pressure that individuals feel on whether to report health care fraud. PBC refers to the degree of difficulty an individual perceives in reporting health care fraud. In addition, this study explores the two dimensions of Perceived Risks (PRISK) and Perceived Consequences (PCONSE). PRISK is respondents’ perceptions of risk after reporting [34], and PCONSE is respondents’ perception of the consequences of health insurance fraud. The theoretical framework is illustrated in Fig. 1.

### Variables

#### Dependent variables

Two different scenarios were constructed to measure individuals’ willingness to report health insurance fraud in both familiar and unfamiliar healthcare settings. Scenario one is “When you find fraudulent insurance practices in hospitals or pharmacies that you frequently visit, are you willing to report?“, scenario two is “When you find fraudulent insurance practices in hospitals and pharmacies that you don’t frequently visit, are you willing to report?“. Since it is difficult to predict when people will get sick, we can’t set a specific frequency for “frequently”. Therefore, we defined “the healthcare providers frequently visited by the public” by asking the respondents, “In your last five visits to a healthcare provider, the place you visited the most times”. In the Chinese healthcare context, due to the behavioral inertia of people visiting the doctor and the implementation of the hierarchical medical system, it is reasonable to assume that the public will frequently visit the same healthcare institution to receive healthcare services, which will make them more familiar with that healthcare setting.

The options for the two questions in both scenarios were set to “not willing at all, not very willing, not sure, very willing, and willing at all”. Similar to other studies [35, 36], in the multiple logistic regression analysis, the dependent variable (willingness to report) was divided into two categories, with 0 indicating unwillingness (including “not willing at all “, “not very willing”, or “not sure”) and 1 indicating willingness (including “very willing” or “willing at all”).

#### Independent variables

##### Socio-demographic characteristics

The socio-demographic characteristics of the participants included gender, age, residency, education level, marital status, and annual household income. We also included respondents’ contact with healthcare providers as socio-demographic variables, including whether they had used health insurance reimbursement in the last three years, and whether they had accompanied others to medical appointments.

##### Behavioral decision variables

Guided by the extended Theory of Planned Behavior, we chose relevant variables to explain the factors affecting the public’s willingness to report, and the codes and descriptions of the variables are shown in Table 2. Except for PCONSE1 (1 = Illegal behavior, 2 = Immoral behavior, 3 = Both have), all variables were measured using a 5-point Likert scale. A score of 3 was used as a medium-level level, above 3 as a high-level group, and below 3 as a low-level group. The Cronbach’s α for all items in Table 2 is 0.715, which is an acceptable level of reliability.

**Table 2:** Behavioral decision variables

Items	Code	Description
Attitudes toward the behavior (ATT)	ATT1	Whether the public perceives that they have the responsibility to report health insurance fraud when they encounter it.
	ATT2	Whether the public perceives their participation in reporting health insurance fraud as effective.
	ATT3	Whether the public supports the whistleblowing behavior of others.
Subjective norm (SN)	SN1	The extent to which the whistleblowing behavior of family members or friends influences the public’s willingness to report health insurance fraud.
	SN2	The extent to which the whistleblowing behavior of experts and academics influences the public’s willingness to report health insurance fraud.
	SN3	The extent to which government disclosure of health insurance fraud cases affects the public’s willingness to report health insurance fraud.
Perceived behavioral control (PBC)	PBC1	The extent to which public health care anti-fraud reporting channels are open to the public.
	PBC2	High or low cost (time, effort, and money) for the public to report health insurance fraud.
	PBC3	The public’s assessment of their ability to participate in reporting health insurance fraud.
Perceived Risks (PRISK)	PRISK1	The extent to which the public are concerned about their safety after reporting health insurance fraud.
	PRISK2	The government will protect the privacy and information security of the whistleblower after the report of health insurance fund anti-fraud.
	PRISK3	Public satisfaction with the protections provided by the government for health fund anti-fraud whistleblowers.
Perceived Consequences (PCONSE)	PCONSE1	Health insurance fraud is illegal or immoral?
	PCONSE2	The extent to which the public perceives health insurance fraud to be common.
	PCONSE3	Public awareness of the severity of the consequences of health insurance fraud.

### Statistical analysis

All statistical analyses were performed using Stata 16.0 and confidence intervals for this study were calculated at the 95% level. All tests were two-tailed, and the statistical significance level was set as *P*-value less than 0.05. Descriptive statistical analysis was used to describe the characteristics of the participants and the individual’s willingness to report in two scenarios of familiar or unfamiliar healthcare settings. McNemar’s test was used to compare the public’s willingness to report under the two scenarios. Chi-square tests were used to analyze the significance of the association between an individual’s willingness to report health insurance fraud and the different categories of independent variables. Multiple logistic regression analysis was conducted to identify the factors influencing the individual’s willingness to report in the two scenarios.

## Results

### Characteristics of participants

Table 3 demonstrates the basic characteristics of the respondents. Of the 828 participants, 61.35% were female, 79.23% were under 45 years old, 85.87% lived in the urban and 72.83% of participants had obtained a university degree or higher. In terms of marriage, 53.86% of the participants were single and 43.12% were married. 50.97% of the respondents had an annual household income greater than RMB 80,000. In the past three years, 37.20% of respondents had experienced health insurance reimbursement, and 84.30% of respondents had experienced accompanying someone to a medical appointment.

**Table 3:** Demographic characteristics of the participants (N = 828)

Characteristics	Respondents	Willingness to report a familiar healthcare setting	*P*-value	Willingness to report an unfamiliar healthcare setting	*P*-value
	N (%)	n (%)		n (%)	
Gender			0.158		0.223
Male	320(38.65)	195(60.94)		168(52.50)	
Female	508(61.35)	335(65.94)		289(56.89)	
Age			0.050		0.047
≤ 44	656(79.23)	431(65.70)		374(57.01)	
45–59	172(20.77)	99(57.56)		83(48.26)	
Residency			0.467		0.089
Urban	711(85.87)	459(64.56)		401(56.40)	
Rural	117(14.13)	71(60.68)		56(47.86)	
Education level			0.018		< 0.001
Elementary school and below	12(1.45)	5(41.67)		1(8.33)	
Junior high and high School	213(25.72)	123(57.75)		101(47.42)	
University and above	603(72.83)	402(66.67)		355(58.87)	
Marital status			0.136		0.496
Single	446(53.86)	292(65.47)		252(56.50)	
Married	357(43.12)	225(63.03)		194(54.34)	
Divorced	22(2.66)	10(45.45)		9(40.91)	
Widowhood	3(0.36)	3(100.00)		2(66.67)	
Annual household income			0.009		0.111
≤ 30,000	106(12.80)	55(51.89)		52(49.06)	
30,001–50,000	125(15.10)	73(58.40)		60(48.00)	
50,001–80,000	175(21.14)	120(68.57)		99(56.57)	
>80,000	422(50.97)	282(66.82)		246(58.29)	
Whether used health insurance reimbursement in the past three years			0.005		0.664
No	520(62.80)	314(60.38)		284(54.62)	
Yes	308(37.20)	216(70.13)		173(56.17)	
Whether accompanied others to medical appointments in the past three years			0.001		0.597
No	130(15.70)	67(51.54)		69(53.08)	
Yes	698(84.30)	463(66.33)		388(55.59)	

### The public’s willingness to report health insurance fraud in different healthcare settings

Table 4 shows the distribution of factors that may be relevant to an individual’s willingness to report in both familiar and unfamiliar healthcare settings. The results show that when health insurance fraud occurs in a familiar healthcare setting, 64.00% of individuals were willing to report it. When health insurance fraud occurred in an unfamiliar setting, 55.19% of individuals were willing to report it (McNemar’s χ²=26.51, *P* < 0.05). As shown in Table 4, in both scenarios, except PCONSE2, respondents’ attitudes toward public participation in reporting health insurance fraud (ATT), subjective norms (SN), perceived behavioral control (PBC), perceived risk after reporting (PRISK), and perceptions of the consequences of health insurance fraud (PCONSE) were all associated with the respondents’ whistleblowing willingness (*P* < 0.05).

**Table 4:** Distribution of factors that may be related to an individual’s willingness to report in two scenarios (N = 828)

Characteristics	Respondents N (%)	Willingness to report a familiar healthcare setting n (%)	*P*-value	Willingness to report an unfamiliar healthcare setting n (%)	*P*-value
Willingness to report	828(100.00)	530(64.00)		457(55.19)	
ATT1			< 0.001		<0.001
No responsibility	79(9.54)	34(43.04)		24(30.38)	
So-so	266(32.13)	130(48.87)		103(38.72)	
Responsibility	483(58.33)	366(75.78)		330(68.32)	
ATT2			< 0.001		<0.001
Not effect	242(29.23)	136(56.20)		115(13.89)	
So-so	367(44.32)	221(26.69)		184(22.22)	
Effect	219(26.45)	173(20.89)		158(19.08)	
ATT3			< 0.001		<0.001
Not supported	26(3.14)	16(61.54)		11(42.31)	
So-so	126(15.22)	34(26.98)		34(26.98)	
Support	676(81.64)	480(71.01)		412(60.95)	
SN1			< 0.001		<0.001
No effect	85(10.27)	50(58.82)		36(42.35)	
So-so	258(31.16)	119(46.12)		106(41.09)	
Effect	485(58.57)	361(74.43)		315(64.95)	
SN2			< 0.001		<0.001
No effect	130(15.70)	71(54.62)		56(43.08)	
So-so	313(37.80)	149(47.60)		124(39.62)	
Effect	385(46.50)	310(80.52)		277(71.95)	
SN3			< 0.001		<0.001
No effect	100(12.08)	50(50.00)		39(39.00)	
So-so	274(33.09)	126(45.99)		108(39.42)	
Effect	454(54.83)	354(77.97)		310(68.28)	
PBC1			< 0.001		<0.001
Not open	197(23.79)	113(57.36)		95(26.39)	
So-so	360(43.48)	198(55.00)		168(61.99)	
Open	271(32.73)	219(80.81)		194(71.59)	
PBC2			< 0.001		0.045
Low cost	97(11.71)	61(62.89)		53(54.64)	
So-so	303(36.59)	167(55.12)		151(49.83)	
High cost	428(51.69)	302(70.56)		253(59.11)	
PBC3			< 0.001		<0.001
Not available	194(23.43)	92(47.42)		79(40.72)	
So-so	359(43.36)	206(57.38)		173(48.19)	
Available	275(33.21)	232(84.36)		205(74.55)	
PRISK1			0.009		0.176
Not concerned	121(14.61)	80(66.12)		73(60.33)	
So-so	154(18.60)	82(53.25)		76(49.35)	
Concerned	553(66.79)	368(66.55)		308(55.70)	
PRISK2			< 0.001		<0.001
Disagree	84(10.14)	40(47.62)		41(48.81)	
So-so	234(28.26)	123(52.56)		97(41.45)	
Agree	510(61.59)	367(71.96)		319(62.55)	
PRISK3			< 0.001		<0.001
Unsatisfied	82(9.90)	35(42.68)		40(48.78)	
So-so	318(38.41)	171(53.77)		132(41.51)	
Satisfied	428(51.69)	324(75.70)		285(66.59)	
PCONSE1			0.001		0.009
Illegal behavior	267(32.25)	167(62.55)		149(55.81)	
Immoral behavior	54(6.52)	23(42.59)		19(35.19)	
Both have	507(61.23)	340(67.06)		289(57.00)	
PCONSE2			0.262		0.798
Uncommon	311(37.56)	210(67.52)		176(56.59)	
So-so	295(35.63)	183(62.03)		159(53.90)	
Common	222(26.81)	137(61.71)		122(54.95)	
PCONSE3			< 0.001		<0.001
Not serious	9(1.09)	4(44.44)		3(33.33)	
So-so	99(11.96)	37(37.37)		37(37.37)	
Serious	720(86.96)	489(67.92)		417(57.92)	

### Factors influencing the public’s willingness to report health insurance fraud in different healthcare settings

Table 5 demonstrates the factors that influence the public’s willingness to report in both familiar and unfamiliar healthcare settings. The results of multiple logistic regression show that when health insurance fraud occurs in a familiar healthcare setting, the public who perceive that individuals have the responsibility to maintain the security of the health insurance fund were more willing to report health insurance fraud compared to those who do not perceive it (*OR* = 1.45, *P* = 0.007).

The public’s ability to report health insurance fraud positively influenced the public’s willingness to report health insurance fraud (*OR* = 1.69, *P* < 0.001). Compared to those who were dissatisfied with the government’s protections for whistleblowers, the higher the public’s satisfaction with the government’s protection of whistleblower privacy and information security, the greater their willingness to report health insurance fraud (*OR* = 1.44, *P* = 0.025). The more serious the consequences caused by health insurance fraud are perceived by individuals, the more willing they are to report it (*OR* = 1.61, *P* = 0.042).

The 4th and 5th columns in Table 5 demonstrate the factors that influence the public’s willingness to report in an unfamiliar setting. In an unfamiliar healthcare setting, the willingness to report health insurance fraud was greater among the public who believed they had a responsibility to maintain the safety of the health insurance fund compared to those who did not (*OR* = 1.78, *P* < 0.001). Individuals’ ability to report health insurance fraud positively influenced the public’s willingness to report health insurance fraud (*OR* = 1.44, *P* = 0.004).

**Table 5:** The factors that influence the public’s willingness to report in a familiar setting (N = 828)

Variables	A familiar healthcare setting		An unfamiliar healthcare setting	
	OR (95% *CI*)	*P*-value	OR (95% *CI*)	*P*-value
Gender	1.29(0.904,1.829)	0.162	1.26(0.901,1.749)	0.179
Age	0.89(0.538,1.474)	0.653	0.99(0.608,1.599)	0.955
Residency	1.15(0.712,1.872)	0.560	0.95(0.599,1.511)	0.833
Education level	1.01(0.651,1.551)	0.982	1.48(0.969,2.248)	0.070
Marital status	0.81(0.557,1.174)	0.265	1.01(0.710,1.435)	0.959
Annual household income	1.17(0.994,1.373)	0.059	1.08(0.921,1.255)	0.356
Whether used health insurance reimbursement in the past three years	1.12(0.783,1.591)	0.545	0.75(0.534,1.044)	0.087
Whether accompanied others to medical appointments in the past three years	1.70(1.086,2.665)	**0.020**	0.89(0.571,1.373)	0.587
ATT1	1.45(1.109,1.902)	**0.007**	1.78(1.363,2.321)	**< 0.001**
ATT2	0.98(0.754,1.264)	0.854	1.05(0.823,1.329)	0.713
ATT3	1.55(1.095,2.200)	**0.013**	1.56(1.099,2.219)	**0.013**
SN1	1.15(0.865,1.522)	0.340	1.26(0.956,1.656)	0.101
SN2	1.18(0.883,1.587)	0.258	1.29(0.975,1.701)	0.075
SN3	1.52(1.126,2.045)	**0.006**	1.35(1.010,1.795)	**0.042**
PBC1	1.03(0.793,1.340)	0.819	1.10(0.859,1.405)	0.454
PBC2	1.24(0.968,1.575)	0.089	0.99(0.788,1.254)	0.958
PBC3	1.69(1.292,2.198)	**0.000**	1.44(1.125,1.853)	**0.004**
PRISK1	1.05(0.828,1.319)	0.710	0.90(0.726,1.124)	0.363
PRISK2	1.14(0.845,1.527)	0.397	1.02(0.767,1.360)	0.886
PRISK3	1.44(1.047,1.995)	**0.025**	1.19(0.872,1.625)	0.272
PCONSE1(reference = Illegal behavior)				
Immoral behavior	0.65(0.318,1.312)	0.227	0.48(0.235,0.982)	**0.044**
Both have	1.06(0.735,1.518)	0.768	0.84(0.595,1.185)	0.319
PCONSE2	0.81(0.650,1.005)	0.055	0.95(0.773,1.164)	0.613
PCONSE3	1.61(1.018,2.555)	**0.042**	1.18(0.750,1.869)	0.469

## Discussion

The main finding of the study was that young and middle-aged adults were more likely to report health insurance fraud in familiar healthcare settings than in unfamiliar ones. Several possible reasons can account for this. Firstly, people have a greater sense of ownership and belonging in familiar scenarios, and their willingness to play the role of active bystander and to report is stronger [37]. Secondly, the Social Preference Model proposes that the public possesses a preference for fair outcomes [38–40]. Most people have relatively high levels of inequality aversion, while moral transgressions in familiar scenarios trigger higher levels of aversion. The combination of these two factors results in a higher willingness to report health insurance fraud in familiar healthcare settings.

The study also found that the public’s perception of their risk after reporting was correlated with the public’s willingness to report health insurance fraud when in a familiar healthcare setting, but not in an unfamiliar one. Numerous studies and practices have shown that most whistleblowers usually experience retaliation from multiple sources, either explicitly or implicitly. Anonymity is critically important and is one of the main ways to protect whistleblowers from and against retaliation [41, 42]. There is a relatively high level of anonymity when the public is in an unfamiliar healthcare setting. Therefore, in an unfamiliar healthcare setting with good anonymity, perceived risks are not a major consideration for the public when reporting health insurance fraud, and the impact of the public’s perceived risks on their willingness to report is not relevant. This finding is confirmed by the findings of Elka Johansson and other scholars [43].

The results of the study also showed that satisfaction with the protective measures taken by the government against whistleblowers was positively correlated with the public’s willingness to report when they were in a familiar healthcare setting. This may be because when individuals are regularly present in a particular location, they meet repeatedly with people within that location, and in the process, familiar strangers emerge, i.e. individuals who never interact with each other but are identifiable with each other, and the anonymity of individuals is reduced. As a result, individuals in a familiar healthcare setting are more likely to be caught in the ‘potential whistleblower’s ethical dilemma’ than those in an unfamiliar healthcare setting [44]. They experience health insurance fraud but do not report it for fear of possible retaliation and the threat. This has been demonstrated in studies by scholars such as Jordan Pappa and Brad Smith [45, 46]. In a familiar healthcare setting, it is therefore the extent to which whistleblowers are protected that is one of the key core factors influencing the public’s willingness to report. The more satisfied the public is with the government’s protective measures for whistleblowers, the more it will help the public overcome moral cowardice and the more willing the public will be to report.

In addition, the study also showed that the public’s willingness to report was significantly related to their perception of the severity of the consequences of health insurance fraud in familiar healthcare settings, while in unfamiliar scenarios, the public’s willingness to report was not related to the dimension. As mentioned above, the public has a higher sense of ownership and a greater aversion to fraud in familiar scenarios than in unfamiliar ones. As a result, the public’s willingness to report health insurance fraud in familiar scenarios is heightened by a trio of psychological factors, including a strong aversion to inequality, a sense of ownership, and a preference for social justice. The experimental findings of Janet P, Jawad Khan, and other scholars have shown that the severity of misconduct is positively correlated with the public’s willingness to report, which is consistent with the findings of this paper [29, 47, 48]. In contrast, the public’s sense of ownership is relatively shallow in unfamiliar healthcare settings, and the severity of health insurance fraud does not trigger a strong sense of unequal aversion among the public, so the public’s perception of the severity of the consequences of health insurance fraud has a relatively weak effect on their willingness to report.

Finally, another important finding of this study suggests that attitudes towards the reporting of health insurance fraud were one of the effective factors influencing the public’s willingness to engage in health insurance anti-fraud. In particular, the public’s willingness to report was stronger when they believed they had a responsibility to safeguard the health insurance fund, regardless of the circumstances. Existing studies point out that the public’s perception of responsibility is positively related to their willingness to report violations [49, 50], with this study being consistent with this finding.

There are some limitations of this study. Firstly, due to the influence of COVID-19, it is difficult to carry out our study in a real healthcare organization, but through the form of the situational hypothesis, which affects the objectivity of the results to a certain extent. Secondly, due to the accessibility of the data, we were unable to directly measure the respondents’ familiarity with the healthcare settings, but instead measured it by whether or not the respondents frequently visited a particular healthcare setting, which is subject to possible error. Thirdly, as research into theoretical models for understanding and studying public intentions moves deeper, future research should use more detailed models that incorporate more factors that influence public behavioral intentions. Last but not least, the study did not explore in depth how the healthcare settings affect the public’s willingness to report, e.g., by analyzing the mechanisms of interaction between the variables of interest and the settings, which will be the focus of our next research.

## Conclusion

This study investigates the differences in the public’s willingness to report health insurance fraud when it occurs in familiar or unfamiliar healthcare settings and the influencing factors. The findings suggest that individuals are more willing to report health insurance fraud in familiar healthcare settings than in unfamiliar ones, where their perception of the severity of the consequences of health insurance fraud and their perceived risk after reporting play an important role. Meanwhile, the public’s sense of responsibility for maintaining the security of the health insurance fund, the disclosure of fraudulent cases by the government, and the perception of their ability to report had a significant effect on their willingness to report, and this effect was independent of the healthcare setting. Therefore, the government’s publicizing of fraud cases and enhancing the public’s sense of responsibility and ability to maintain the safety of the health insurance fund may be the way to increase the public’s willingness to report.

## Data Availability

The datasets generated and analyzed during the current study are not publicly available because the datasets are currently used for another project, but are available from the corresponding author on reasonable request.
